# Transcriptome analysis of fibroblasts from schizophrenia patients reveals differential expression of schizophrenia-related genes

**DOI:** 10.1038/s41598-020-57467-z

**Published:** 2020-01-20

**Authors:** Mitra Etemadikhah, Adnan Niazi, Lennart Wetterberg, Lars Feuk

**Affiliations:** 1 Department of Immunology, Genetics and Pathology, Science for Life Laboratory Uppsala, Uppsala University, Uppsala, Sweden; 2 Department of Clinical Neuroscience (CNS), K8, Karolinska Institute, Stockholm, Sweden

**Keywords:** Gene expression, Neurodevelopmental disorders

## Abstract

Schizophrenia is a complex neurodevelopmental disorder with high rate of morbidity and mortality. While the heritability rate is high, the precise etiology is still unknown. Although schizophrenia is a central nervous system disorder, studies using peripheral tissues have also been established to search for patient specific biomarkers and to increase understanding of schizophrenia etiology. Among all peripheral tissues, fibroblasts stand out as they are easy to obtain and culture. Furthermore, they keep genetic stability for long period and exhibit molecular similarities to cells from nervous system. Using a unique set of fibroblast samples from a genetically isolated population in northern Sweden, we performed whole transcriptome sequencing to compare differentially expressed genes in seven controls and nine patients. We found differential fibroblast expression between cases and controls for 48 genes, including eight genes previously implicated in schizophrenia or schizophrenia related pathways; *HGF*, *PRRT2*, *EGR1*, *EGR3*, *C11orf87*, *TLR3, PLEKHH2* and *PIK3CD*. Weighted gene correlation network analysis identified three differentially co-expressed networks of genes significantly-associated with schizophrenia. All three modules were significantly suppressed in patients compared to control, with one module highly enriched in genes involved in synaptic plasticity, behavior and synaptic transmission. In conclusion, our results support the use of fibroblasts for identification of differentially expressed genes in schizophrenia and highlight dysregulation of synaptic networks as an important mechanism in schizophrenia.

## Introduction

Schizophrenia is a neurodevelopmental disorder with lifetime risk of about 1%^1,2^. Due to the high rate of morbidity and mortality, schizophrenia is classified as a severe psychiatric disorder^2,3^. It is thought to be initiated by brain development disruption triggered by genetics, or environment, or both^4^. The heritability of schizophrenia has been estimated to up to 80%^5,6^, and over the past decade genetic studies have started to elucidate the complex genetic etiology of schizophrenia. Genome-wide association studies (GWAS) and structural variation studies have given rise to numerous findings of genetic contribution^7^. The largest GWAS study to date resulted in more than 100 distinct associated loci, with the majority not previously identified^7–9^. Genomic studies have also identified rare recurrent copy number variants (CNVs) which contribute to schizophrenia at high risk^10–13^. Similarly, whole genome sequencing (WGS) and exome sequencing have identified enrichment of de novo mutations and rare disruptive variants in genes belonging to specific neurodevelopmental pathways^7,14^. One large transcriptome study together with genetic data also provided further insight for potential underlying mechanisms of three major psychiatric disorders including schizophrenia^15^. The genetic data thus indicate both common variants conferring low risk and rare variants of strong effect.

Although schizophrenia is considered a central nervous system disorder, studies using peripheral tissues in search for disease-associated biomarkers have also been established. Identification of biomarkers that are specific to patients and can differentiate patients from controls with high sensitivity can lead to better understanding of central- and peripheral-related features. This is of importance also at the clinical level since it can be very beneficial for diagnostics and prognostics, and also providing better treatments for these patients^16–18^. Among all peripheral tissues, fibroblasts stand out as they are readily available, easy to culture under controlled conditions, and also showed similar expression profiles to post mortem brain in several studies of psychiatric disorders^19–21^. Using non-neuronal cells as a proxy to investigate the pathophysiology of neurodevelopmental disorders has long been established. Relevant biochemical processes can be investigated easier in peripheral tissues. Many neurodevelopmental disorders are caused by dysregulation of genes and pathways expressed also in fibroblasts or even ubiquitously. As an example, defects in basic molecular mechanisms like DNA damage and repair is common across all cell types^22^.

Other options for studies of brain related disorders include animal models, which may shed light on brain structure and function alterations and also behavioral disturbance^23–25^. However, since the disorder is unique to humans not all symptoms, such as hallucinations, are reproducible in animals and not all symptoms observed in animals are fully reflected in humans^26^. Using brain biopsies in neuropsychiatric disorders is another option but it also comes with its own challenges. Factors such as individual lifestyle, history of drug abuse and medications, cause of death, and postmortem interval can affect the findings and make their interpretation more complicated^27^. Furthermore, post-mortem biopsies cannot be used for diagnostic and therapeutic approaches^28^.

Taking all these limitations together, the interest in using patient-derived peripheral tissues, especially human dermal fibroblasts, in psychiatric disorders has increased over the last decades. Fibroblasts are easy to obtain, culture, and store. They keep genetic stability for up to 15–20 passages^29^. They have molecular similarities with cells from the central nervous system^28^, and also express transcription factors, receptors and signaling pathways similar to neurons to a high extent^30^. Fibroblasts can be used to establish induced pluripotent stem cells (iPSCs), which can then be differentiated into neurons. This has successfully been used as a model system to study schizophrenia^31^. On the other hand, as the cells grow on an artificial surface, they differ from neurons in some aspects including lack of *in vivo* signals, synapse formation, neurotransmitter release, and ion channel expression^28,32^. All these features establish fibroblasts as a complementary cell model to post-mortem and animal studies of neuropsychiatric disorders.

A classical approach to study genetic diseases is the analysis of large pedigrees with many affected members. Collection and analysis of such pedigrees was attempted in early days of psychiatric genetic research, focusing on linkage studies^33,34^. However, these samples have often not been analyzed using modern genomic approaches. Increased prevalence of schizophrenia in large pedigrees or isolated regions may be due to an increased burden of common risk factors, but could also be explained by one or a few strong risk factors. Analysis of large pedigrees may provide an alternative for identification of rare risk factors or disrupted signaling pathways.

Several epidemiological studies in northern Sweden have reported high prevalence of neuropsychiatric and neurodevelopmental disorders, including schizophrenia^35^. Previous studies have also described isolated regions with prevalence of psychiatric disorders several times higher than in the general Swedish population^36–38^. One of these genetically isolated regions with an increased prevalence of schizophrenia has been studied using various approaches ranging from epidemiology to genetics over the last 60 years^37^. The first founders of the population were three closely related Finnish families who settled in the region in early 17th century^37^.

In this study, we aimed to identify genes or gene networks associated with schizophrenia in a unique set of fibroblast samples from the genetically isolated region in northern Sweden, where the population has been shown to exhibit an increased prevalence of schizophrenia^37^. We sequenced RNA extracted from cultured fibroblasts from 16 individuals, seven controls and nine patients to compare differentially expressed genes. Data was analyzed for differential gene expression and co-expression networks were then constructed using weighted gene correlation network analysis (WGCNA)^39^.

## Results

### Identification of differentially expressed genes

In total, 48 DEGs with adjusted p-value < 0.1 were identified in patients compared to controls (Supplementary Table S2). Among these 48 DEGs, eight genes were identified in previous studies of schizophrenia or schizophrenia related pathways (Table 1): *HGF*, *PRRT2*, *EGR1*, *EGR3*, *C11orf87*, *TLR3, PLEKHH2* and *PIK3CD* (Fisher exact test, P = 0.00021).

**Table 1:** Differentially-expressed genes that have overlap with other schizophrenia-related studies (Fisher exact test, p-value = 0.00021).

Ensemble Gene ID	Gene Symbol	Log2Fold Change	P-value	adj. P-value	Main finding	Study type
ENSG00000019991	*HGF*	3.81	9.27E-07	0.004	Reported upregulation in schizophrenia by CMC^40^	Expression
ENSG00000167371	*PRRT2*	1.38	3.75E-05	0.031	CNVs at this gene genomic position have been associated with schizophrenia^48,49^	Genetic
ENSG00000120738	*EGR1*	1.36	6.24E-05	0.045	First reported to be associated with schizophrenia by PGC^8^ Reported upregulation in schizophrenia^51^	Genetic Expression
ENSG00000171608	*PIK3CD*	−0.79	6.23E-05	0.045	Evidence for link between the NRG1-ErbB4 signaling pathway and PIK3CD in schizophrenia^70^	Expression
ENSG00000185742	*C11orf87*	1.00	6.77E-05	0.046	First reported to be associated with schizophrenia by PGC^8^	Genetic
ENSG00000164342	*TLR3*	2.34	0.00022	0.09	Reported de novo missense mutations associated with schizophrenia^62^	Genetic
ENSG00000152527	*PLEKHH2*	1.79	0.00026	0.097	Reported de novo missense mutations in schizophrenia patients^67^	Genetic
ENSG00000179388	*EGR3*	2.72	0.00025	0.097	Reported SNPs associated with schizophrenia^56–59^	Genetic

CMC: CommonMind Consortium; PGC: Psychiatric Genomics Consortium.

### Co-expression network analysis

To study networks of genes that were expressed together, we further analyzed our data with WGCNA. Using the R library WGCNA to construct the co-expression networks, 32 modules were detected in total. Using a p-value cut-off of <0.05 for condition (affected vs unaffected) three modules were identified as significant: module skyblue with 176 genes, module floralwhite with 79 genes, and module lightcyan with 293 genes in total (Supplementary Fig. S1). Of these three modules, MEskyblue also showed significant results for age.

**Figure 1:**
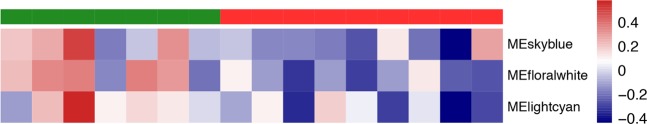
The eigengene co-expression modules heatmap. Top legend: cases are colored in red and controls in green. Side legend: eigengene values showing the direction of co-expression in modules.

To investigate the overall expression profile of the genes in each module, the eigengene was calculated. All these three modules, MEskyblue, MEfloralwhite, and MElightcyan were generally downregulated in patient fibroblasts (red) compared to controls (green) (Fig. 1).

The genes identified in each module are listed in Supplementary Table S3.

### Gene ontology and functional enrichment

**Figure 2:**
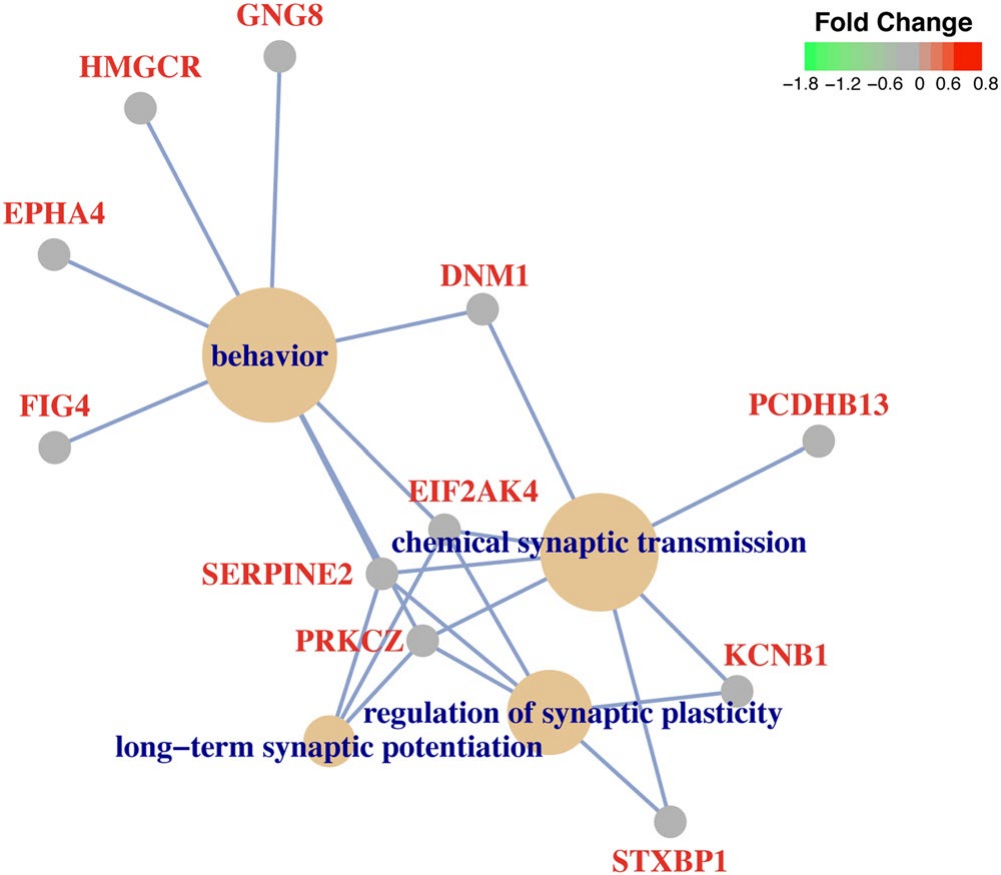
Top 4 enriched biological pathways in the floralwhite module and the genes associated with each GO category.

We further performed functional enrichment analysis to investigate if co-expressed genes in these three modules were significantly enriched in certain biological pathways relevant to schizophrenia pathophysiology. Module skyblue had positive regulation of axon extension as the most significant GO category, including the genes *FN1*, *MACF1*, *NDEL1* and *SEMA7A*. Module floralwhite was highly enriched in biological pathways including regulation of synaptic plasticity, behavior and synaptic transmission. The top four enriched biological pathways of floralwhite module and their associated genes have been illustrated in Fig. 2. Supplementary Table S4 shows the top enriched biological pathways in each module.

## Discussion

In this study we used fibroblasts collected from a unique population in northern Sweden. The genetically isolated region has been the focus of studies on neuropsychiatric and neurodevelopmental disorders since more than 50 years^37^, due to an increased prevalence of neurodevelopmental disorders in the region. The increased genetic risk is presumed to be explained by a small founder population carrying multiple risk variants. Genetic analyses have shown that the inheritance is complex and not limited to one strong risk factor^37^. Over the last decades, increased mobility in and out of the previously isolated region has led to a reduction in risk for schizophrenia and developmental disorders. The fibroblasts used in the current study were initially collected in 1978 making them a unique resource for analysis of schizophrenia. A drawback of using this resource is that the number of available samples is highly limited and we recognize that the limited sample size lowers the power to detect subtle changes in expression.

In this transcriptome study, we found differential fibroblast expression between cases and controls for 48 genes, including eight genes previously implicated in schizophrenia. The genes *HGF*, *PRRT2*, *EGR1*, *EGR3*, *C11orf87*, *TLR3* and *PLEKHH2* have been reported in either genetic analysis or gene expression analysis of schizophrenia, and were all upregulated in fibroblasts from patients in our study. In addition, we found downregulation of *PIK3CD*, also previously associated with schizophrenia.

Hepatocyte growth factor (*HGF*), located within the genomic position 7q21.11, showed the largest fold change and was upregulated in cases versus controls. The upregulation of *HGF* in schizophrenia affected individuals was also reported in the CommonMind Consortium (CMC) study^40^. Hepatocyte growth factor/scatter factor (HGF/SF) is a key factor in proper migration of interneurons from ganglionic eminence (GE) to cerebral cortex^41^. HGF/SF displays differentiation and mitogenic activities by signaling through its receptor, MET, in neuronal^42,43^ and non-neuronal tissues^44,45^.

The expression of *PRRT2*, located within the genomic position 16p11.2 microduplication region, was also elevated significantly in cases versus controls. Several studies implicated the association of recurrent structural mutations in this genomic hotspot in individuals with schizophrenia. Mutations at 16p11.2 cause high risk of schizophrenia and microduplication of this region is associated with an 8–24 folds increased risk of the disorder^10,12,46–50^.

Early growth response 1 (*EGR1*) is located within the genomic position 5q31.2 and *C11orf87* within 11q22.3. These loci were first reported to be associated with schizophrenia by the Schizophrenia Working Group of the Psychiatric Genomics Consortium in 2014^8^. *EGR1* was also reported as a potential biomarker to differentiate major psychoses, as its upregulation in fibroblasts and peripheral blood cells was specific to schizophrenia compared to Major Depressive Disorder (MDD) and Bipolar Disorder (BD)^51^. *EGR1*, a part of the immediate early genes (IEGs) family, is an early response gene to different growth stimuli^52^. It is involved in neuronal plasticity pathway though the precise underlying mechanism is still unknown^53,54^. Early growth response 3 (*EGR3*), located within genomic position 8p21.3, is another member of IEGs family and its expression is regulated downstream of neuregulin 1 (NRG1) signaling cascade in some cell types^55^. Single nucleotide polymorphisms (SNPs) in *EGR3* in different populations of Japanese, Korean, Han Chinese, and European origin have been previously reported in association with schizophrenia^56–59^. In our study, we observed upregulation of *EGR3* in schizophrenia patients compared to controls.

The *C11orf87*, also known as neuronal integral membrane protein 1 (NEURIM1) is primarily expressed in brain tissue. It is the only gene present within a locus (chr11:109285471-109610071) identified to harbor variation associated with schizophrenia in several genome-wide association studies^8,60^ in different populations, as well as association with self-reported educational attainment^61^.

One study in 2014, using exome sequencing of 623 schizophrenia trios, implicated Toll-like receptor 3 (*TLR3*) de novo missense mutation associated with schizophrenia^62^. *TLR3* is located within the genomic position 4q35.1. Viral infection during pregnancy is associated with increased risk of neuropsychiatric disorders including schizophrenia in offspring^63,64^. Exposing pregnant mice to a synthetic virus led to inhibited cortical neurogenesis and behavioral abnormalities in offspring in a *TLR3* dependent manner and through activation of innate immunity^65^. Another study using cultured neurons and mouse brain showed that TLR3 suppresses disrupted in schizophrenia 1 (*Disc1*) expression and consequently neuronal development^66^.

In an exome sequencing study of 42 sporadic and 15 familial schizophrenia trios, de novo missense SNV of *PLEKHH2*, located at genomic position 2p21, in an individual diagnosed with schizophrenia was ranked in the top 15% of probable haploinsufficient genes among candidate mutations based on functional impact^67^.

We also identified downregulation of phosphatidylinositol 3-kinase catalytic delta (*PIK3CD*), located at genomic position 1p36.22, among DEGs in patients versus controls. The signaling pathway of NRG1 and its receptor ErbB4 plays an important role in neural development and synaptic plasticity^68^ and has been associated with schizophrenia before^69^. One study in 2012, provides evidence for link between the NRG1-ErbB4 signaling pathway and PIK3CD in schizophrenia. They also describe PIK3CD as a potential therapeutic target for psychiatric disorders. Using a mouse model in their study to inhibit PIK3CD, they could block the effect of amphetamine in the animal and reverse the schizophrenia-related behaviors^70^.

We also applied WGCNA to identify differentially co-expressed networks of genes. As a result, differential expression in three modules were detected as significantly associated with schizophrenia. All three modules were significantly suppressed in patients compared to control, and one module (floralwhite) was highly enriched of genes involved in synaptic plasticity, behavior, synaptic transmission, GABAergic pathways. All these pathways and their underlying mechanisms have been implicated in schizophrenia before^62^. The critical role of synaptic plasticity in learning, memory, and also formation of mature neural circuits has made some scientists to think of synaptic plasticity as a key pathogenic feature in schizophrenia^71^. Post-mortem^72,73^ and large-scale genetic studies^13,74^ have constantly shown synaptic plasticity impairment in schizophrenia patients compared to controls. Additionally, cognitive functions including working memory are mediated by dorsolateral prefrontal cortex and, as a core feature of schizophrenia, have been impaired in patients^75,76^. This impairment is partly due to abnormalities in GABA-mediated circuitry^77,78^. A recent genetic analysis of another large pedigree from Northern Sweden showed that the members had an increased risk for schizophrenia based on polygenic risk scores, but did not carry rare variants contributing significant increase in risk^38^. Our results support a similar etiology in the present pedigree, where we find general support for modules with genes previously implicated in schizophrenia by both genetic and expression studies, but no single genes that stand out as rare and strong contributors to risk.

Our results support the use of fibroblast cell lines as an important resource for studies of schizophrenia. Although fibroblasts can never represent the diversity and complexity of brain tissue, many pathways of importance in neuropsychiatric disorders are expressed. We show that relevant pathways are detected also in this limited sample size. Previous studies of fibroblasts from schizophrenia patients and controls have shown mixed results, with one study found several differentially expressed genes (including EGR1 mentioned above) and two other studies finding no differentially expressed genes^79,80^. It is possible that the genetically isolated population from where these samples were collected represents a less heterogeneous sample compared to a general European population sample, and this homogeneity may have increased our ability to detect relevant gene expression differences between patient and control samples.

The cell lines used in this study were collected in 1978, and this has to be taken into consideration as a potential limitation to the study. According to published literatures the actual time of storage in liquid nitrogen has very limited impact, while number of passages and freeze/thaw cycles play a larger role. In one study where blood samples were stored at −80 °C and in liquid nitrogen separately and each for the maximum of 19 years, there was no systematic effect of the storage time on RNA quality^81^. This finding was supported by another study where RNA extracted from endocrine tissue stored at −80 °C for 27 years has been assessed based on RNA integrity number (RIN)^82^. In 2019, the Ontario Tumour Bank also published a report of tissue quality control over 11 years of storage in liquid nitrogen. Their results show no time-dependent decline in sample quality, and they concluded that extended periods of storage at cryogenic temperature is suitable for sample preservation in biobanks^83^. The fibroblasts used here have all gone through a very limited number of passages and have been stored under optimal conditions. All samples were collected in the same year and there has been no difference in sample handling between patient and control cell lines. We note that fibroblasts are also a valuable resource as they may differentiate into neuronal cells, either directly or via de-differentiation into stem cells. This would represent an interesting avenue forward for these cell lines in order to understand the role of the differentially expressed genes in an even more relevant cell model.

In conclusion, we performed transcriptome study of fibroblasts from patients and controls collected in a region with increased prevalence of schizophrenia. Our results support the findings of several genes previously implicated in schizophrenia and highlight dysregulation of synaptic networks as an important mechanism in schizophrenia. The results further lend support to the collection and use of fibroblasts as an important resource for studies of neuropsychiatric disorders.

## Materials and Methods

### Clinical materials

The donors of the fibroblast samples included in this study belong to three very large, interrelated pedigrees originated from Finnish ancestors^37^. All patients fulfilled the criteria for schizophrenia according to DSM-III-R, as well as according to^84^ and^85^. Healthy individuals included in this study have been followed up to age 65 and beyond for evaluation of psychiatric health problems. Skin biopsies were collected for establishment of primary fibroblast cultures in 1978. Doctor Jan Arvid Böök, performed the skin biopsies in the patient’s home. The patients and their healthy control parents and siblings were generally examined on the same day. A limited portion of the skin on the person’s forearm was first anesthetized. A punch blade, which was attached to a pencil-like handle, was rotated through the skin to the subcutaneous fat layer, giving a cylindrical tissue core. Minimal bleeding was noted and the area was left to heal without any complications. The skin samples were immediately sent from North Sweden to Berta Santesson at Uppsala University for culturing of fibroblasts. Divided portions of the fibroblast cultures were frozen on liquid nitrogen over the years until a small portion of the specific samples were thawed for the present study.

In this study, fibroblast cultures from 16 individuals including seven healthy and nine schizophrenics were analyzed. Supplementary Table S1 shows the demographic data of all 16 individuals. All samples were collected with informed consent and in accordance with the Declaration of Helsinki. The study has been approved by the Regional Ethical Review Board in Uppsala with approval dnr 2014/263. All research was performed in accordance with relevant regulations.

### Sample preparation

Fibroblast cells were maintained in high glucose DMEM medium (Sigma) supplemented with 10% fetal bovine serum (FBS), 1% MEM non-essential amino acids, 2 mM of L-glutamine and 1× penicillin/streptomycin (Sigma) under optimal conditions, 37 °C and 5% CO_2_. When they became confluent, the cells were split to larger culture flasks. All fibroblasts were cultured just one more passage after thaw and were homogenized in trizol (TRI) for RNA extraction afterward. Total RNA was extracted from homogenized cells using Ribopure kit (Ambion) and treated with TURBO DNase (Ambion) according to the manufacturer’s instructions.

### RNA sequencing

Sequencing libraries for 16 individual samples were prepared from 180–280 ng total RNA using the TruSeq stranded mRNA library preparation kit (Cat# RS-122- 2101/2102, Illumina Inc.) including polyA selection according to the manufacturer’s protocol. Sequencing was then performed on HiSeq. 2500 machine by the SNP&SEQ Technology Platform in Uppsala and data from three lanes of sequencing was uploaded on Uppmax.

### Reads mapping and differential expression analysis

RNAseq data containing adapter sequences and low-quality reads were filtered out with Trimmomatic 0.32^86^. Filtered reads were mapped to human reference genome hg19 with STAR 2.5.1b software^87^, with an average of 29 million uniquely mapped reads per sample. Genes annotated in ENSEMBL annotation v75 were quantified with python framework HTSeq. 0.6.1^88^. Reads mapping to multiple positions in the genome were not counted. Next, reads mapping to technical replicates were summed gene wise. After normalization, genes with <5 reads in 40% of the samples were removed. DESeq. 2 v1.16.1 was used for differential expression analysis^89^. After differential expression analysis, the resulting p-values were adjusted for multiple testing using the Benjamini–Hochberg procedure.

### Co-expression network analysis

Co-expression networks were constructed using the R library WGCNA^39^. Briefly, the analysis was performed using same samples and number of genes that were used for differential expression analysis. Following this the DESeq. 2 implementation of variance stabilizing transform was used on the read counts for each gene. To find the correct power to use when constructing the network, soft thresholding powers for one to 20 were calculated using a signed network. As RNA sequencing data are likely to contain outliers the bi-weight mid-correlation was used^90^. After reviewing the resulting scale free topology index for the powers one to 20, a power of four was selected as this power gave the best tradeoff between network free topology and connectivity. A signed hybrid co-expression network was then calculated using standard settings. Modules were identified from the network and the eigenvectors of each module were correlated with the variables age, passage, sex, and condition (patient or control).

### Gene ontology enrichment analysis

GO enrichment was performed on the genes with significant differential expression using an R package GOseq, controlling for gene length biases^91^. As background for the GO analysis all genes where DESeq2 performed a test for differential expression were used. Functional annotation enrichment was also performed for the genes resulting from WGCNA analysis. Overlaps with different gene sets for each significant module was assessed using Fisher’s exact test. The genes in each module were used to define a gene set, and each such gene set was tested for overlap with the gene set of DEGs in our fibroblast study, 693 DEGs in schizophrenia from the CommonMind Consortium (CMC)^40^, and curated genetic associations with schizophrenia^8,48,49,62,67,92,93^. Briefly, the genetic associations with schizophrenia were derived from: 108 discovered in a common variant GWAS^8^, 12 CNVs^49^, 756 nonsynonymous *de novo* mutations and 114 rare variants with loss of function, collated from several studies^48,62,67,92,93^, and are available at CMC knowledge portal (http://commonmind.org). A literature review of the differentially expressed genes was also performed to investigate their link to schizophrenia in previous studies.

## Supplementary information

Supplementary Information.

## Data Availability

The datasets generated during and/or analyzed during the current study are available from the corresponding author on reasonable request.
