# Supplementary material for: Transcriptome analysis of fibroblasts from schizophrenia patients reveals differential expression of schizophrenia-related genes
Source: Sci Rep. 2020 Jan 20;10:630. doi: 10.1038/s41598-020-57467-z (PMC6971273; doi:10.1038/s41598-020-57467-z)
Supplement: Supplementary file 1 — Supplementary Information. [file 41598_2020_57467_MOESM1_ESM.pdf]

Transcriptome analysis of fibroblasts from schizophrenia patients reveals differential expression of schizophrenia-related genes

Mitra Etemadikhah[1], Adnan Niazi[1], Lennart Wetterberg[2] and Lars Feuk[1*]

[1] Department of Immunology, Genetics and Pathology, Science for Life Laboratory Uppsala, Uppsala University, Uppsala, Sweden

[2] Department of Clinical Neuroscience (CNS), K8, Karolinska Institute, Stockholm, Sweden

[*]Corresponding author:

Lars Feuk

Department of Immunology, Genetics and Pathology

Box 815, BMC B11:4, Uppsala University

751 08 Uppsala, Sweden

Phone: +46 18 471 4827

E-mail: Lars.Feuk@igp.uu.se

Supplementary Table S1: An overview of demographic data

Demographics	Schizophrenia (n=9)	Control (n=7)	Statistics
Sex Female/Male	5F:4M	5F:2M	0.89 (Chi-square)
Age of disease onset in years	19.2 ± 4.3	-	N/A
Age at which skin biopsy has been taken	32 ± 7.9	39.9 ± 20.03	0.36 (t-test)

Supplementary Table S2: Differentially Expressed Genes (DEGs) in Fibroblasts

Row.names	baseMean	log2FoldChange	pvalue	padj	gene_symbol	biotype
ENSG00000149289	1263.35509	0.736095003	7.52E-11	1.20E-06	ZC3H12C	protein_coding
ENSG00000146592	188.821104	2.265212252	1.12E-08	8.93E-05	CREB5	protein_coding
ENSG00000168528	4591.60472	-1.758075961	3.39E-08	0.00018002	SERINC2	protein_coding
ENSG00000019991	343.364144	3.805874858	9.27E-07	0.00369518	HGF	protein_coding
ENSG00000198300	28.4400347	-3.898520087	1.22E-06	0.00388696	PEG3	protein_coding
ENSG00000149596	2486.84723	-1.228427868	2.41E-06	0.00641517	JPH2	protein_coding
ENSG00000095587	140.572923	-1.929432049	4.87E-06	0.01109035	TLL2	protein_coding
ENSG00000173917	1088.30708	-0.717016853	5.68E-06	0.01131029	HOXB2	protein_coding
ENSG00000120075	202.344579	-1.531928462	7.91E-06	0.01401423	HOXB5	protein_coding
ENSG00000144659	1746.79882	-0.379662502	1.32E-05	0.02063359	SLC25A38	protein_coding
ENSG00000198919	1020.21433	0.519038008	1.42E-05	0.02063359	DZIP3	protein_coding
ENSG00000105538	76.6113255	-4.799819369	1.90E-05	0.02329886	RASIP1	protein_coding
ENSG00000126016	1069.36779	1.297043505	1.86E-05	0.02329886	AMOT	protein_coding
ENSG00000070182	400.308931	-1.789812347	2.28E-05	0.02424519	SPTB	protein_coding
ENSG00000171368	20.716006	-1.596892742	2.23E-05	0.02424519	TPPP	protein_coding
ENSG00000173253	27.6030636	3.849836806	2.87E-05	0.02847318	DMRT2	protein_coding
ENSG00000178202	8170.91388	0.508928906	3.04E-05	0.02847318	KDELC2	protein_coding
ENSG00000167371	274.831657	1.380727819	3.75E-05	0.03146678	PRRT2	protein_coding
ENSG00000259820	114.708105	0.87352013	3.69E-05	0.03146678	AC083843.1	lincRNA
ENSG00000149212	1215.68296	1.047109946	5.11E-05	0.04075616	SESN3	protein_coding
ENSG00000120738	2802.99039	1.361000693	6.24E-05	0.04522384	EGR1	protein_coding
ENSG00000171608	1016.93485	-0.78865285	6.23E-05	0.04522384	PIK3CD	protein_coding
ENSG00000144583	6118.03896	-0.879725264	6.96E-05	0.04619857	04-Mar	protein_coding
ENSG00000185742	1596.17422	1.00304696	6.77E-05	0.04619857	C11orf87	protein_coding
ENSG00000108821	483429.712	-0.741685563	0.000104	0.06631578	COL1A1	protein_coding
ENSG00000115317	1624.48588	-0.37347778	0.00010939	0.06706662	HTRA2	protein_coding
ENSG00000123610	174.388568	2.196452627	0.00011738	0.06929964	TNFAIP6	protein_coding
ENSG00000005020	1832.41146	0.432451092	0.00014143	0.08051659	SKAP2	protein_coding

ENSG00000065809	1893.78943	1.242388597	0.000153	0.08130024	FAM107B	protein_coding
ENSG00000205683	364.09917	1.224383073	0.00014927	0.08130024	DPF3	protein_coding
ENSG00000101040	1696.89753	0.352230089	0.00016445	0.08300528	ZMYND8	protein_coding
ENSG00000196611	31558.4872	2.644646924	0.00016662	0.08300528	MMP1	protein_coding
ENSG00000169744	2666.65096	1.063645586	0.00017767	0.08582654	LDB2	protein_coding
ENSG00000124406	45.1183596	1.467493731	0.00022265	0.09045698	ATP8A1	protein_coding
ENSG00000130517	952.60076	-1.034746439	0.00022543	0.09045698	PGPEP1	protein_coding
ENSG00000157557	3517.19104	0.785239453	0.00021239	0.09045698	ETS2	protein_coding
ENSG00000164342	27.6953704	2.336562236	0.0002174	0.09045698	TLR3	protein_coding
ENSG00000168906	13290.4895	0.502722867	0.00021003	0.09045698	MAT2A	protein_coding
ENSG00000182372	1364.21051	-0.717335667	0.00020076	0.09045698	CLN8	protein_coding
ENSG00000273247	54.9895546	1.049087175	0.00022698	0.09045698	RP11-83A24.2	antisense
ENSG00000152527	134.227439	1.79403818	0.00025506	0.09680782	PLEKHH2	protein_coding
ENSG00000179388	284.785384	2.718632158	0.00025257	0.09680782	EGR3	protein_coding
ENSG00000082153	12435.1883	0.368768949	0.00027359	0.09730222	BZW1	protein_coding
ENSG00000155980	77.7326412	1.918079817	0.00026413	0.09730222	KIF5A	protein_coding
ENSG00000198121	4759.81556	0.608206999	0.00027468	0.09730222	LPAR1	protein_coding
ENSG00000168575	5031.46371	-0.800672727	0.0002834	0.09821078	SLC20A2	protein_coding
ENSG00000251281	50.9036573	-3.394732976	0.00029033	0.0984708	CTD-2066L21.2	lincRNA
ENSG00000118785	722.433157	-2.62828212	0.00029833	0.09907588	SPP1	protein_coding

SupplementaryTable S3: List of Genes in Module Skyblue

ensid	gene_symbol	biotype
ENSG00000031698	SARS	protein_coding
ENSG00000040341	STAU2	protein_coding
ENSG00000047346	FAM214A	protein_coding
ENSG00000054793	ATP9A	protein_coding
ENSG00000058729	RIOK2	protein_coding
ENSG00000059728	MXD1	protein_coding
ENSG00000070669	ASNS	protein_coding
ENSG00000071575	TRIB2	protein_coding
ENSG00000072422	RHOBTB1	protein_coding
ENSG00000076043	REXO2	protein_coding
ENSG00000077713	SLC25A43	protein_coding
ENSG00000082196	C1QTNF3	protein_coding
ENSG00000089177	KIF16B	protein_coding
ENSG00000091513	TF	protein_coding
ENSG00000097033	SH3GLB1	protein_coding
ENSG00000101290	CDS2	protein_coding
ENSG00000102053	ZC3H12B	protein_coding
ENSG00000102452	NALCN	protein_coding
ENSG00000102893	PHKB	protein_coding
ENSG00000102931	ARL2BP	protein_coding
ENSG00000103160	HSDL1	protein_coding
ENSG00000104154	SLC30A4	protein_coding
ENSG00000104490	NCALD	protein_coding
ENSG00000106688	SLC1A1	protein_coding
ENSG00000108821	COL1A1	protein_coding
ENSG00000109016	DHRS7B	protein_coding
ENSG00000110768	GTF2H1	protein_coding
ENSG00000111911	HINT3	protein_coding
ENSG00000112425	EPM2A	protein_coding
ENSG00000113163	COL4A3BP	protein_coding
ENSG00000113273	ARSB	protein_coding
ENSG00000114850	SSR3	protein_coding
ENSG00000114942	EEF1B2	protein_coding
ENSG00000115414	FN1	protein_coding
ENSG00000116138	DNAJC16	protein_coding
ENSG00000116954	RRAGC	protein_coding
ENSG00000118363	SPCS2	protein_coding
ENSG00000122550	KLHL7	protein_coding
ENSG00000123739	PLA2G12A	protein_coding
ENSG00000124215	CDH26	protein_coding
ENSG00000125633	CCDC93	protein_coding
ENSG00000126775	ATG14	protein_coding
ENSG00000127472	PLA2G5	protein_coding
ENSG00000127603	MACF1	protein_coding

ENSG00000129055	ANAPC13	protein_coding
ENSG00000129493	HEATR5A	protein_coding
ENSG00000130703	OSBPL2	protein_coding
ENSG00000130997	POLN	protein_coding
ENSG00000131379	C3orf20	protein_coding
ENSG00000134375	TIMM17A	protein_coding
ENSG00000134851	TMEM165	protein_coding
ENSG00000135069	PSAT1	protein_coding
ENSG00000135823	STX6	protein_coding
ENSG00000136881	BAAT	protein_coding
ENSG00000137942	FNBP1L	protein_coding
ENSG00000138119	MYOF	protein_coding
ENSG00000138623	SEMA7A	protein_coding
ENSG00000138758	11-Sep	protein_coding
ENSG00000139998	RAB15	protein_coding
ENSG00000141298	SSH2	protein_coding
ENSG00000142188	TMEM50B	protein_coding
ENSG00000143164	DCAF6	protein_coding
ENSG00000143199	ADCY10	protein_coding
ENSG00000143248	RGS5	protein_coding
ENSG00000143643	TTC13	protein_coding
ENSG00000144366	GULP1	protein_coding
ENSG00000145349	CAMK2D	protein_coding
ENSG00000148444	COMMD3	protein_coding
ENSG00000151092	NGLY1	protein_coding
ENSG00000151388	ADAMTS12	protein_coding
ENSG00000152785	BMP3	protein_coding
ENSG00000156804	FBXO32	protein_coding
ENSG00000157734	SNX22	protein_coding
ENSG00000161217	PCYT1A	protein_coding
ENSG00000162298	SYVN1	protein_coding
ENSG00000162614	NEXN	protein_coding
ENSG00000162627	SNX7	protein_coding
ENSG00000163513	TGFBR2	protein_coding
ENSG00000163754	GYG1	protein_coding
ENSG00000164144	ARFIP1	protein_coding
ENSG00000165476	REEP3	protein_coding
ENSG00000166167	BTRC	protein_coding
ENSG00000166579	NDEL1	protein_coding
ENSG00000166971	AKTIP	protein_coding
ENSG00000167110	GOLGA2	protein_coding
ENSG00000169570	DTWD2	protein_coding
ENSG00000171291	ZNF439	protein_coding
ENSG00000173261	PLAC8L1	protein_coding
ENSG00000173482	PTPRM	protein_coding
ENSG00000176171	BNIP3	protein_coding

ENSG00000176732	PFN4	protein_coding
ENSG00000178033	FAM26E	protein_coding
ENSG00000179889	PDXDC1	protein_coding
ENSG00000180479	ZNF571	protein_coding
ENSG00000181852	RNF41	protein_coding
ENSG00000182568	SATB1	protein_coding
ENSG00000182700	IGIP	protein_coding
ENSG00000182901	RGS7	protein_coding
ENSG00000185532	PRKG1	protein_coding
ENSG00000186567	CEACAM19	protein_coding
ENSG00000186889	TMEM17	protein_coding
ENSG00000188993	LRRC66	protein_coding
ENSG00000196502	SULT1A1	protein_coding
ENSG00000196693	ZNF33B	protein_coding
ENSG00000197149	RP11-452G18.2	pseudogene
ENSG00000197296	FITM2	protein_coding
ENSG00000197647	ZNF433	protein_coding
ENSG00000197694	SPTAN1	protein_coding
ENSG00000197702	PARVA	protein_coding
ENSG00000197826	C4orf22	protein_coding
ENSG00000197885	NKIRAS1	protein_coding
ENSG00000197894	ADH5	protein_coding
ENSG00000198001	IRAK4	protein_coding
ENSG00000198105	ZNF248	protein_coding
ENSG00000198429	ZNF69	protein_coding
ENSG00000199266	SNORA60	snoRNA
ENSG00000203876	RP11-451M19.3	protein_coding
ENSG00000204177	BMS1P1	pseudogene
ENSG00000213626	LBH	protein_coding
ENSG00000213976	CTD-2561J22.2	pseudogene
ENSG00000213996	TM6SF2	protein_coding
ENSG00000214113	LYRM4	protein_coding
ENSG00000214269	LGMNP1	pseudogene
ENSG00000214366	RP11-138H14.1	pseudogene
ENSG00000215105	TTC3P1	pseudogene
ENSG00000219986	BTF3P7	pseudogene
ENSG00000220793	RPL21P119	pseudogene
ENSG00000221051	AC118344.1	miRNA
ENSG00000221930	FAM45B	pseudogene
ENSG00000224597	PTCHD3P1	antisense
ENSG00000225241	RP11-640M9.2	pseudogene
ENSG00000225975	AC074138.3	lincRNA
ENSG00000226381	RP11-119F19.2	antisense
ENSG00000228399	RP4-575N6.2	pseudogene
ENSG00000228998	RP11-697E2.7	pseudogene
ENSG00000230069	LRRC37A15P	pseudogene

ENSG00000230204	FTH1P5	pseudogene
ENSG00000230658	KLHL7-AS1	lincRNA
ENSG00000230701	FBXW4P1	pseudogene
ENSG00000231007	CDC20P1	pseudogene
ENSG00000232224	LINC00202-1	lincRNA
ENSG00000232273	FTH1P1	pseudogene
ENSG00000233225	AC004987.9	pseudogene
ENSG00000234933	CDC42P1	pseudogene
ENSG00000234981	RP11-534L20.4	pseudogene
ENSG00000235174	RPL39P3	pseudogene
ENSG00000235513	RP4-756G23.5	antisense
ENSG00000239521	GATS	pseudogene
ENSG00000240225	ZNF542	pseudogene
ENSG00000240854	RP11-64K7.1	pseudogene
ENSG00000240857	RDH14	protein_coding
ENSG00000241962	C2orf15	protein_coding
ENSG00000242095	RN7SL113P	misc_RNA
ENSG00000244567	AC096772.6	lincRNA
ENSG00000246263	KB-431C1.4	antisense
ENSG00000250067	YJEFN3	protein_coding
ENSG00000251396	RP11-163N6.2	lincRNA
ENSG00000253797	UTP14C	protein_coding
ENSG00000254272	RP11-382J24.2	pseudogene
ENSG00000254363	CTB-131B5.5	processed_transcript
ENSG00000255633	MTRNR2L9	protein_coding
ENSG00000260787	RP11-797A18.4	lincRNA
ENSG00000261101	RP4-545K15.5	sense_overlapping
ENSG00000267213	AC007773.2	antisense
ENSG00000267242	AC069278.4	lincRNA
ENSG00000268816	CTD-3093M3.1	lincRNA
ENSG00000268894	PLCE1-AS1	antisense
ENSG00000268913	AC026806.2	lincRNA
ENSG00000271043	MTRNR2L2	protein_coding
ENSG00000271452	RP11-342K6.2	sense_intronic
ENSG00000271553	RP11-274B21.10	lincRNA
ENSG00000272091	RP4-758J24.5	lincRNA
ENSG00000272452	RP11-391M1.4	lincRNA
ENSG00000272551	RP11-324L17.1	lincRNA
ENSG00000272732	RP5-1159O4.2	lincRNA
ENSG00000272947	RP11-71H17.9	lincRNA

List of Genes in Module Floralwhite

ensid	gene_symbol	biotype
ENSG00000067606	PRKCZ	protein_coding
ENSG00000068793	CYFIP1	protein_coding
ENSG00000076344	RGS11	protein_coding
ENSG00000081177	EXD2	protein_coding
ENSG00000106479	ZNF862	protein_coding
ENSG00000106976	DNM1	protein_coding
ENSG00000107185	RGP1	protein_coding
ENSG00000107960	OBFC1	protein_coding
ENSG00000110025	SNX15	protein_coding
ENSG00000112367	FIG4	protein_coding
ENSG00000112972	HMGCS1	protein_coding
ENSG00000113161	HMGCR	protein_coding
ENSG00000116106	EPHA4	protein_coding
ENSG00000124523	SIRT5	protein_coding
ENSG00000128829	EIF2AK4	protein_coding
ENSG00000131779	PEX11B	protein_coding
ENSG00000134897	BIVM	protein_coding
ENSG00000134899	ERCC5	protein_coding
ENSG00000135622	SEMA4F	protein_coding
ENSG00000135919	SERPINE2	protein_coding
ENSG00000136854	STXBP1	protein_coding
ENSG00000137177	KIF13A	protein_coding
ENSG00000142583	SLC2A5	protein_coding
ENSG00000144815	NXPE3	protein_coding
ENSG00000147099	HDAC8	protein_coding
ENSG00000147174	ACRC	protein_coding
ENSG00000151338	MIPOL1	protein_coding
ENSG00000151694	ADAM17	protein_coding
ENSG00000154917	RAB6B	protein_coding
ENSG00000158406	HIST1H4H	protein_coding
ENSG00000158445	KCNB1	protein_coding
ENSG00000163644	PPM1K	protein_coding
ENSG00000164188	RANBP3L	protein_coding
ENSG00000166323	C11orf65	protein_coding
ENSG00000167414	GNG8	protein_coding
ENSG00000177599	ZNF491	protein_coding
ENSG00000178386	ZNF223	protein_coding
ENSG00000180867	PDIA3P1	pseudogene
ENSG00000181227	RP4-682C21.2	pseudogene
ENSG00000184277	TM2D3	protein_coding
ENSG00000184530	C6orf58	protein_coding
ENSG00000184678	HIST2H2BE	protein_coding
ENSG00000186026	ZNF284	protein_coding
ENSG00000187372	PCDHB13	protein_coding
ENSG00000196975	ANXA4	protein_coding
ENSG00000197050	ZNF420	protein_coding

ENSG00000198945	L3MBTL3	protein_coding
ENSG00000200090	Y_RNA	misc_RNA
ENSG00000204272	RP11-622K12.1	processed_transcript
ENSG00000206052	DOK6	protein_coding
ENSG00000213139	CRYGS	protein_coding
ENSG00000215504	AP000974.1	protein_coding
ENSG00000221866	PLXNA4	protein_coding
ENSG00000225334	RP11-449J1.1	antisense
ENSG00000225830	ERCC6	protein_coding
ENSG00000226549	SCDP1	pseudogene
ENSG00000228121	PHBP3	pseudogene
ENSG00000228938	SNRPD2P1	pseudogene
ENSG00000230177	RP5-1112D6.4	antisense
ENSG00000230606	AC159540.1	lincRNA
ENSG00000230665	PRKRIRP1	pseudogene
ENSG00000234817	RP3-400B16.1	lincRNA
ENSG00000235106	LINC00094	antisense
ENSG00000236349	SUCLG2P2	pseudogene
ENSG00000237133	AC020594.5	antisense
ENSG00000242159	ABCF2P1	pseudogene
ENSG00000242173	ARHGDIG	protein_coding
ENSG00000243927	MRPS6	protein_coding
ENSG00000244701	RP5-894A10.2	antisense
ENSG00000250878	METTL21EP	pseudogene
ENSG00000257151	PWAR6	lincRNA
ENSG00000257594	GALNT4	protein_coding
ENSG00000261087	KB-1460A1.5	lincRNA
ENSG00000263002	ZNF234	protein_coding
ENSG00000267370	CTD-2623N2.3	pseudogene
ENSG00000269292	CTB-12A17.3	antisense
ENSG00000269946	RP11-2B6.3	lincRNA
ENSG00000271270	TMCC1-AS1	antisense
ENSG00000273243	CTA-217C2.2	lincRNA

List of Genes in Module Lightcyan

ensid	gene_symbol	biotype
ENSG00000001617	SEMA3F	protein_coding
ENSG00000002746	HECW1	protein_coding
ENSG00000007237	GAS7	protein_coding
ENSG00000011275	RNF216	protein_coding
ENSG00000022567	SLC45A4	protein_coding
ENSG00000042286	AIFM2	protein_coding
ENSG00000046647	GEMIN8	protein_coding
ENSG00000047578	KIAA0556	protein_coding
ENSG00000051620	HEBP2	protein_coding
ENSG00000060656	PTPRU	protein_coding
ENSG00000065989	PDE4A	protein_coding
ENSG00000075240	GRAMD4	protein_coding
ENSG00000087157	PGS1	protein_coding
ENSG00000088543	C3orf18	protein_coding
ENSG00000089163	SIRT4	protein_coding
ENSG00000089505	CMTM1	protein_coding
ENSG00000091986	CCDC80	protein_coding
ENSG00000095321	CRAT	protein_coding
ENSG00000099377	HSD3B7	protein_coding
ENSG00000099910	KLHL22	protein_coding
ENSG00000099953	MMP11	protein_coding
ENSG00000100246	DNAL4	protein_coding
ENSG00000100316	RPL3	protein_coding
ENSG00000100577	GSTZ1	protein_coding
ENSG00000102032	RENBP	protein_coding
ENSG00000102981	PARD6A	protein_coding
ENSG00000102984	ZNF821	protein_coding
ENSG00000103091	WDR59	protein_coding
ENSG00000103264	FBXO31	protein_coding
ENSG00000103343	ZNF174	protein_coding
ENSG00000103742	IGDCC4	protein_coding
ENSG00000104365	IKBKB	protein_coding
ENSG00000105204	DYRK1B	protein_coding
ENSG00000105281	SLC1A5	protein_coding
ENSG00000105327	BBC3	protein_coding
ENSG00000106404	CLDN15	protein_coding
ENSG00000106624	AEBP1	protein_coding
ENSG00000106948	AKNA	protein_coding
ENSG00000107819	SFXN3	protein_coding
ENSG00000113296	THBS4	protein_coding
ENSG00000114626	ABTB1	protein_coding
ENSG00000114841	DNAH1	protein_coding
ENSG00000116213	WRAP73	protein_coding
ENSG00000118971	CCND2	protein_coding
ENSG00000119943	PYROXD2	protein_coding
ENSG00000121753	BAI2	protein_coding

ENSG00000122515	ZMIZ2	protein_coding
ENSG00000126882	FAM78A	protein_coding
ENSG00000127364	TAS2R4	protein_coding
ENSG00000127366	TAS2R5	protein_coding
ENSG00000127507	EMR2	protein_coding
ENSG00000129933	MAU2	protein_coding
ENSG00000130164	LDLR	protein_coding
ENSG00000130649	CYP2E1	protein_coding
ENSG00000131043	AAR2	protein_coding
ENSG00000131797	CLUHP3	pseudogene
ENSG00000132376	INPP5K	protein_coding
ENSG00000132405	TBC1D14	protein_coding
ENSG00000132510	KDM6B	protein_coding
ENSG00000132613	MTSS1L	protein_coding
ENSG00000132694	ARHGEF11	protein_coding
ENSG00000132801	ZSWIM3	protein_coding
ENSG00000132881	RSG1	protein_coding
ENSG00000133247	SUV420H2	protein_coding
ENSG00000133619	KRBA1	protein_coding
ENSG00000133943	C14orf159	protein_coding
ENSG00000134574	DDB2	protein_coding
ENSG00000135148	TRAFD1	protein_coding
ENSG00000135596	MICAL1	protein_coding
ENSG00000135709	KIAA0513	protein_coding
ENSG00000135736	CCDC102A	protein_coding
ENSG00000136059	VILL	protein_coding
ENSG00000136732	GYPC	protein_coding
ENSG00000138829	FBN2	protein_coding
ENSG00000138942	RNF185	protein_coding
ENSG00000139200	PIANP	protein_coding
ENSG00000139428	MMAB	protein_coding
ENSG00000139974	SLC38A6	protein_coding
ENSG00000140044	JDP2	protein_coding
ENSG00000140459	CYP11A1	protein_coding
ENSG00000140931	CMTM3	protein_coding
ENSG00000141837	CACNA1A	protein_coding
ENSG00000141934	PPAP2C	protein_coding
ENSG00000143258	USP21	protein_coding
ENSG00000143353	LYPLAL1	protein_coding
ENSG00000143847	PPFIA4	protein_coding
ENSG00000143867	OSR1	protein_coding
ENSG00000144214	LYG1	protein_coding
ENSG00000144567	FAM134A	protein_coding
ENSG00000145692	BHMT	protein_coding
ENSG00000145916	RMND5B	protein_coding
ENSG00000145919	BOD1	protein_coding
ENSG00000145949	MYLK4	protein_coding
ENSG00000145982	FARS2	protein_coding

ENSG00000146733	PSPH	protein_coding
ENSG00000147403	RPL10	protein_coding
ENSG00000148356	LRSAM1	protein_coding
ENSG00000148399	DPH7	protein_coding
ENSG00000148541	FAM13C	protein_coding
ENSG00000149257	SERPINH1	protein_coding
ENSG00000149273	RPS3	protein_coding
ENSG00000150594	ADRA2A	protein_coding
ENSG00000151468	CCDC3	protein_coding
ENSG00000151572	ANO4	protein_coding
ENSG00000151846	PABPC3	protein_coding
ENSG00000152475	ZNF837	protein_coding
ENSG00000152804	HHEX	protein_coding
ENSG00000153786	ZDHHC7	protein_coding
ENSG00000153902	LGI4	protein_coding
ENSG00000154240	CEP112	protein_coding
ENSG00000154767	XPC	protein_coding
ENSG00000155511	GRIA1	protein_coding
ENSG00000156345	CDK20	protein_coding
ENSG00000157613	CREB3L1	protein_coding
ENSG00000157823	AP3S2	protein_coding
ENSG00000160172	FAM86C2P	pseudogene
ENSG00000160216	AGPAT3	protein_coding
ENSG00000160271	RALGDS	protein_coding
ENSG00000161243	FBXO27	protein_coding
ENSG00000161277	THAP8	protein_coding
ENSG00000161642	ZNF385A	protein_coding
ENSG00000162191	UBXN1	protein_coding
ENSG00000162396	PARS2	protein_coding
ENSG00000162981	FAM84A	protein_coding
ENSG00000163472	TMEM79	protein_coding
ENSG00000164627	KIF6	protein_coding
ENSG00000164638	SLC29A4	protein_coding
ENSG00000164898	C7orf55	protein_coding
ENSG00000165949	IFI27	protein_coding
ENSG00000166123	GPT2	protein_coding
ENSG00000166169	POLL	protein_coding
ENSG00000166289	PLEKHF1	protein_coding
ENSG00000167291	TBC1D16	protein_coding
ENSG00000167522	ANKRD11	protein_coding
ENSG00000167566	NCKAP5L	protein_coding
ENSG00000167716	WDR81	protein_coding
ENSG00000167968	DNASE1L2	protein_coding
ENSG00000167969	ECI1	protein_coding
ENSG00000167972	ABCA3	protein_coding
ENSG00000168096	ANKS3	protein_coding
ENSG00000168672	FAM84B	protein_coding
ENSG00000168904	LRRC28	protein_coding

ENSG00000171703	TCEA2	protein_coding
ENSG00000172053	QARS	protein_coding
ENSG00000172273	HINFP	protein_coding
ENSG00000172350	ABCG4	protein_coding
ENSG00000172530	BANP	protein_coding
ENSG00000172602	RND1	protein_coding
ENSG00000172935	MRGPRF	protein_coding
ENSG00000173548	SNX33	protein_coding
ENSG00000174137	FAM53A	protein_coding
ENSG00000174748	RPL15	protein_coding
ENSG00000175048	ZDHHC14	protein_coding
ENSG00000175764	TTLL11	protein_coding
ENSG00000175886	RPL7AP66	pseudogene
ENSG00000177030	DEAF1	protein_coding
ENSG00000177045	SIX5	protein_coding
ENSG00000177692	DNAJC28	protein_coding
ENSG00000178078	STAP2	protein_coding
ENSG00000178301	AQP11	protein_coding
ENSG00000178623	GPR35	protein_coding
ENSG00000179406	LINC00174	lincRNA
ENSG00000179743	RP11-169K16.9	antisense
ENSG00000181035	SLC25A42	protein_coding
ENSG00000181097	RP11-429J17.2	antisense
ENSG00000181481	RNF135	protein_coding
ENSG00000181982	CCDC149	protein_coding
ENSG00000182272	B4GALNT4	protein_coding
ENSG00000183111	ARHGEF37	protein_coding
ENSG00000184845	DRD1	protein_coding
ENSG00000185924	RTN4RL1	protein_coding
ENSG00000186635	ARAP1	protein_coding
ENSG00000186834	HEXIM1	protein_coding
ENSG00000187535	IFT140	protein_coding
ENSG00000187609	EXD3	protein_coding
ENSG00000187626	ZKSCAN4	protein_coding
ENSG00000188000	OR7D2	protein_coding
ENSG00000188315	C3orf62	protein_coding
ENSG00000188687	SLC4A5	protein_coding
ENSG00000188747	NOXA1	protein_coding
ENSG00000189227	C15orf61	protein_coding
ENSG00000196267	ZNF836	protein_coding
ENSG00000196663	TECPR2	protein_coding
ENSG00000196700	ZNF512B	protein_coding
ENSG00000196814	MVB12B	protein_coding
ENSG00000197681	TBC1D3	protein_coding
ENSG00000197747	S100A10	protein_coding
ENSG00000197766	CFD	protein_coding
ENSG00000197818	SLC9A8	protein_coding
ENSG00000198590	C3orf35	protein_coding

ENSG00000198691	ABCA4	protein_coding
ENSG00000198728	LDB1	protein_coding
ENSG00000198873	GRK5	protein_coding
ENSG00000198915	RASGEF1A	protein_coding
ENSG00000204628	GNB2L1	protein_coding
ENSG00000205090	TMEM240	protein_coding
ENSG00000205663	RP11-706O15.5	lincRNA
ENSG00000206113	RP11-503N18.1	lincRNA
ENSG00000212123	PRR22	protein_coding
ENSG00000213380	COG8	protein_coding
ENSG00000213553	RPLP0P6	pseudogene
ENSG00000213609	RP11-170M17.2	pseudogene
ENSG00000213672	NCKIPSD	protein_coding
ENSG00000213862	CTD-2270N23.1	pseudogene
ENSG00000215190	LINC00680	processed_transcript
ENSG00000215305	VPS16	protein_coding
ENSG00000215790	SLC35E2	protein_coding
ENSG00000215910	C1orf167	protein_coding
ENSG00000217648	RP1-95L4.4	pseudogene
ENSG00000218052	ADAMTS7P4	pseudogene
ENSG00000219755	RP1-199J3.5	pseudogene
ENSG00000220472	RP11-69L16.5	pseudogene
ENSG00000221926	TRIM16	protein_coding
ENSG00000222482	AC005071.1	miRNA
ENSG00000223855	AC147651.3	lincRNA
ENSG00000225051	HMGB3P22	pseudogene
ENSG00000225096	XXbac-BPG55C20.7	lincRNA
ENSG00000225259	ST13P6	pseudogene
ENSG00000225447	RPS15AP10	pseudogene
ENSG00000225568	RP11-384B12.3	pseudogene
ENSG00000225806	RP1-309F20.3	processed_transcript
ENSG00000226742	HSBP1L1	protein_coding
ENSG00000226920	RP5-1068B5.3	lincRNA
ENSG00000227603	RP11-165J3.6	antisense
ENSG00000227973	PIN4P1	pseudogene
ENSG00000228107	AP000692.9	sense_overlapping
ENSG00000228172	RP1-317E23.3	antisense
ENSG00000228224	NACAP1	pseudogene
ENSG00000228331	RPL17P43	pseudogene
ENSG00000228801	RP11-110G21.1	antisense
ENSG00000228878	AC007551.3	lincRNA
ENSG00000229119	CTB-63M22.1	pseudogene
ENSG00000229833	PET100	protein_coding
ENSG00000230832	RP11-325P15.2	pseudogene
ENSG00000232295	RP11-154D6.1	antisense
ENSG00000232385	RPS3AP25	pseudogene
ENSG00000232561	GTF2IP1	pseudogene
ENSG00000233859	ADH5P4	pseudogene

ENSG00000234648	AL162151.3	pseudogene
ENSG00000235649	MXRA5P1	pseudogene
ENSG00000235776	AC000089.3	pseudogene
ENSG00000236432	AC097662.2	antisense
ENSG00000236735	RPL31P63	pseudogene
ENSG00000236976	RP11-165H4.2	pseudogene
ENSG00000237214	RP11-12M9.3	pseudogene
ENSG00000237991	RPL35P1	pseudogene
ENSG00000238082	AC009948.7	pseudogene
ENSG00000239704	CDRT4	protein_coding
ENSG00000239789	MRPS17	protein_coding
ENSG00000242071	RPL7AP6	pseudogene
ENSG00000243364	EFNA4	protein_coding
ENSG00000243710	WDR65	protein_coding
ENSG00000247271	ZBED5-AS1	antisense
ENSG00000248309	MEF2C-AS1	antisense
ENSG00000249142	CTD-2290P7.1	pseudogene
ENSG00000249465	RBMXP4	pseudogene
ENSG00000250327	RP11-173M11.2	pseudogene
ENSG00000250571	GLI4	protein_coding
ENSG00000251085	RP11-893F2.6	lincRNA
ENSG00000251485	AC068134.10	pseudogene
ENSG00000253392	AC006277.2	antisense
ENSG00000254206	NPIPB11	protein_coding
ENSG00000254409	RP11-613D13.4	sense_overlapping
ENSG00000255967	RP11-438L7.3	pseudogene
ENSG00000258982	RP11-638I2.4	antisense
ENSG00000259088	CTD-2017C7.2	antisense
ENSG00000259305	ZHX1-C8ORF76	protein_coding
ENSG00000259673	IQCH-AS1	lincRNA
ENSG00000259807	RP11-426C22.4	lincRNA
ENSG00000260086	RP11-42I10.1	lincRNA
ENSG00000260296	RP11-395I6.3	sense_overlapping
ENSG00000260461	RP11-541N10.3	sense_overlapping
ENSG00000260495	RP11-55K13.1	antisense
ENSG00000261126	RBFADN	processed_transcript
ENSG00000261159	RP11-723O4.9	lincRNA
ENSG00000261168	RP11-68I18.10	sense_overlapping
ENSG00000263422	AC005702.1	miRNA
ENSG00000265458	RP13-20L14.6	antisense
ENSG00000266777	SH3GL1P1	pseudogene
ENSG00000266876	RP11-1148O4.1	pseudogene
ENSG00000266968	RP11-116O18.1	sense_intronic
ENSG00000267288	RP13-890H12.2	antisense
ENSG00000267317	CTB-25B13.12	processed_transcript
ENSG00000268856	AP001579.1	protein_coding
ENSG00000271151	RP11-394I13.2	lincRNA
ENSG00000271912	RP11-661A12.14	lincRNA

ENSG00000272017	RP1-199J3.7	antisense
ENSG00000272112	CTB-113P19.5	lincRNA
ENSG00000272692	RP11-399K21.14	lincRNA
ENSG00000272842	RP11-513M16.7	antisense
ENSG00000272950	RP11-307C18.1	antisense
ENSG00000273230	RP11-1246C19.1	lincRNA
ENSG00000273356	RP11-804H8.6	lincRNA

Supplementary Table S4: GO Analysis_ Module Skyblue

GO.category	GO.over_represented_pvalue	GO.numDEInCat	GO.numInCat	GO.term	GO.ontology
GO:0045773	0.000270903	4	36	positive regulation of axon extension : BP	BP
GO:0051179	0.000533571	55	4204	localization : BP	BP
GO:0046471	0.000973213	3	22	phosphatidylglycerol metabolic process : BP	BP
GO:0035356	0.001192203	2	6	cellular triglyceride homeostasis : BP	BP
GO:0090066	0.001217499	10	352	regulation of anatomical structure size : BP	BP
GO:0048102	0.001640934	2	7	autophagic cell death : BP	BP
GO:0036149	0.001648499	2	7	phosphatidylinositol acyl-chain remodeling : BP	BP
GO:0042149	0.00192514	3	28	cellular response to glucose starvation : BP	BP
GO:0010663	0.002093637	2	8	positive regulation of striated muscle cell apoptotic process : BP	BP
GO:0010666	0.002093637	2	8	positive regulation of cardiac muscle cell apoptotic process : BP	BP
GO:0006810	0.002147037	44	3306	transport : BP	BP
GO:0036148	0.002166927	2	8	phosphatidylglycerol acyl-chain remodeling : BP	BP
GO:0036150	0.002185999	2	8	phosphatidylserine acyl-chain remodeling : BP	BP
GO:0050772	0.002491372	4	64	positive regulation of axonogenesis : BP	BP
GO:0048588	0.002537741	6	155	developmental cell growth : BP	BP
GO:0065008	0.003624319	34	2426	regulation of biological quality : BP	BP
GO:0032535	0.003671617	8	281	regulation of cellular component size : BP	BP
GO:0048639	0.004116446	5	119	positive regulation of developmental growth : BP	BP
GO:0009069	0.004147781	3	36	serine family amino acid metabolic process : BP	BP
GO:0090161	0.004198778	2	11	Golgi ribbon formation : BP	BP
GO:0051234	0.004217341	44	3418	establishment of localization : BP	BP
GO:0010508	0.004939344	4	77	positive regulation of autophagy : BP	BP
GO:0030516	0.005045934	4	78	regulation of axon extension : BP	BP
GO:0010876	0.005661346	7	239	lipid localization : BP	BP
GO:0010506	0.005881593	7	241	regulation of autophagy : BP	BP
GO:0036152	0.005921105	2	13	phosphatidylethanolamine acyl-chain remodeling : BP	BP
GO:0016239	0.006101639	3	42	positive regulation of macroautophagy : BP	BP
GO:0031669	0.006199016	5	130	cellular response to nutrient levels : BP	BP
GO:0010661	0.00670932	2	14	positive regulation of muscle cell apoptotic process : BP	BP
GO:0097320	0.006823593	2	14	membrane tubulation : BP	BP
GO:0050482	0.006829419	2	14	arachidonic acid secretion : BP	BP
GO:1903963	0.006829419	2	14	arachidonate transport : BP	BP
GO:0050770	0.006835778	5	134	regulation of axonogenesis : BP	BP
GO:0007033	0.007617843	6	192	vacuole organization : BP	BP
GO:0006516	0.007859665	2	15	glycoprotein catabolic process : BP	BP
GO:0061387	0.008068352	4	89	regulation of extent of cell growth : BP	BP
GO:0046470	0.008205371	3	46	phosphatidylcholine metabolic process : BP	BP
GO:0038044	0.008216246	1	1	transforming growth factor-beta secretion : BP	BP
GO:1904235	0.008216246	1	1	regulation of substrate-dependent cell migration, cell attachment to substrate : BP	BP
GO:1904237	0.008216246	1	1	positive regulation of substrate-dependent cell migration, cell attachment to substrate : BP	BP
GO:2001201	0.008216246	1	1	regulation of transforming growth factor-beta secretion : BP	BP
GO:2001202	0.008216246	1	1	negative regulation of transforming growth factor-beta secretion : BP	BP
GO:0044710	0.008255634	39	3029	single-organism metabolic process : BP	BP
GO:0007040	0.008440933	3	47	lysosome organization : BP	BP
GO:0080171	0.008440933	3	47	lytic vacuole organization : BP	BP
GO:1902618	0.008675447	1	1	cellular response to fluoride : BP	BP
GO:0006914	0.008750361	9	393	autophagy : BP	BP
GO:1902203	0.008818628	1	1	negative regulation of hepatocyte growth factor receptor signaling pathway : BP	BP
GO:2001112	0.008818628	1	1	regulation of cellular response to hepatocyte growth factor stimulus : BP	BP
GO:2001113	0.008818628	1	1	negative regulation of cellular response to hepatocyte growth factor stimulus : BP	BP
GO:0036296	0.008840101	2	16	response to increased oxygen levels : BP	BP
GO:0055093	0.008840101	2	16	response to hyperoxia : BP	BP
GO:0036151	0.008918668	2	16	phosphatidylcholine acyl-chain remodeling : BP	BP
GO:1903778	0.008983064	1	1	protein localization to vacuolar membrane : BP	BP
GO:0002514	0.009016431	1	1	B cell tolerance induction : BP	BP
GO:0002649	0.009016431	1	1	regulation of tolerance induction to self antigen : BP	BP
GO:0002651	0.009016431	1	1	positive regulation of tolerance induction to self antigen : BP	BP
GO:0002661	0.009016431	1	1	regulation of B cell tolerance induction : BP	BP
GO:0002663	0.009016431	1	1	positive regulation of B cell tolerance induction : BP	BP
GO:0042883	0.009024842	1	1	cysteine transport : BP	BP
GO:1903712	0.009024842	1	1	cysteine transmembrane transport : BP	BP
GO:0002152	0.009042873	1	1	bile acid conjugation : BP	BP
GO:0070165	0.009051179	1	1	positive regulation of adiponectin secretion : BP	BP
GO:0045081	0.009065586	1	1	negative regulation of interleukin-10 biosynthetic process : BP	BP
GO:0061580	0.00906907	1	1	colon epithelial cell migration : BP	BP
GO:0070981	0.009069247	1	1	L-asparagine biosynthetic process : BP	BP
GO:0070982	0.009069247	1	1	L-asparagine metabolic process : BP	BP
GO:0009267	0.00933495	4	92	cellular response to starvation : BP	BP
GO:0003148	0.009791134	2	17	outflow tract septum morphogenesis : BP	BP
GO:0060351	0.009947398	2	17	cartilage development involved in endochondral bone morphogenesis : BP	BP

GO Analysis_ Module Floralwhite

GO.category	GO.over_represented_pvalue	GO.numDEInCat	GO.numInCat	GO.term	GO.ontology
GO:0048167	4.9909E-05	5	100	regulation of synaptic plasticity : BP	BP
GO:0007610	8.84588E-05	8	358	behavior : BP	BP
GO:0060291	0.000437358	3	37	long-term synaptic potentiation : BP	BP
GO:0007268	0.00056616	7	359	chemical synaptic transmission : BP	BP
GO:0098916	0.00056616	7	359	anterograde trans-synaptic signaling : BP	BP
GO:0099536	0.00056616	7	359	synaptic signaling : BP	BP
GO:0099537	0.00056616	7	359	trans-synaptic signaling : BP	BP
GO:0044708	0.000634082	6	262	single-organism behavior : BP	BP
GO:1903035	0.000708776	3	43	negative regulation of response to wounding : BP	BP
GO:0050804	0.00095456	5	188	modulation of synaptic transmission : BP	BP
GO:0045956	0.001026562	2	12	positive regulation of calcium ion-dependent exocytosis : BP	BP
GO:0010664	0.001269142	2	13	negative regulation of striated muscle cell apoptotic process : BP	BP
GO:1990138	0.001305626	4	120	neuron projection extension : BP	BP
GO:0055094	0.001721165	2	15	response to lipoprotein particle : BP	BP
GO:0032101	0.002021399	7	441	regulation of response to external stimulus : BP	BP
GO:0009416	0.002079514	5	222	response to light stimulus : BP	BP
GO:0060292	0.002099277	2	17	long term synaptic depression : BP	BP
GO:0021952	0.0022985	2	18	central nervous system projection neuron axonogenesis : BP	BP
GO:0051259	0.00235734	6	334	protein oligomerization : BP	BP
GO:0048710	0.002364306	2	18	regulation of astrocyte differentiation : BP	BP
GO:0051932	0.002590544	2	19	synaptic transmission, GABAergic : BP	BP
GO:0010665	0.00265932	2	19	regulation of cardiac muscle cell apoptotic process : BP	BP
GO:0010656	0.002686947	2	19	negative regulation of muscle cell apoptotic process : BP	BP
GO:0010662	0.002955298	2	20	regulation of striated muscle cell apoptotic process : BP	BP
GO:0007616	0.003140352	2	21	long-term memory : BP	BP
GO:0050709	0.0031634	3	72	negative regulation of protein secretion : BP	BP
GO:0048588	0.003374084	4	155	developmental cell growth : BP	BP
GO:0006295	0.003392329	2	21	nucleotide-excision repair, DNA incision, 3'-to lesion : BP	BP
GO:0007270	0.003409453	3	75	neuron-neuron synaptic transmission : BP	BP
GO:0008045	0.003456337	2	22	motor neuron axon guidance : BP	BP
GO:0050806	0.003536942	3	76	positive regulation of synaptic transmission : BP	BP
GO:0008299	0.0037274	2	22	isoprenoid biosynthetic process : BP	BP
GO:0021955	0.003742114	2	23	central nervous system neuron axonogenesis : BP	BP
GO:0039520	0.003843408	1	1	induction by virus of host autophagy : BP	BP
GO:0010659	0.003892092	2	23	cardiac muscle cell apoptotic process : BP	BP
GO:0009645	0.004016651	1	1	response to low light intensity stimulus : BP	BP
GO:0046690	0.004016651	1	1	response to tellurium ion : BP	BP
GO:0021793	0.004061413	1	1	chemorepulsion of branchiomotor axon : BP	BP
GO:0015755	0.004129487	1	1	fructose transport : BP	BP
GO:0032445	0.004129487	1	1	fructose import : BP	BP
GO:0097319	0.004129487	1	1	carbohydrate import into cell : BP	BP
GO:0098704	0.004129487	1	1	carbohydrate import across plasma membrane : BP	BP
GO:1990539	0.004129487	1	1	fructose import across plasma membrane : BP	BP
GO:0042628	0.004161096	1	1	mating plug formation : BP	BP
GO:0044704	0.004161096	1	1	single-organism reproductive behavior : BP	BP
GO:0045297	0.004161096	1	1	post-mating behavior : BP	BP
GO:0061108	0.004161096	1	1	seminal vesicle epithelium development : BP	BP
GO:0036046	0.004173478	1	1	protein demalonylation : BP	BP
GO:0036047	0.004173478	1	1	peptidyl-lysine demalonylation : BP	BP
GO:0036048	0.004173478	1	1	protein desuccinylation : BP	BP
GO:0036049	0.004173478	1	1	peptidyl-lysine desuccinylation : BP	BP
GO:0061698	0.004173478	1	1	protein deglutarylation : BP	BP
GO:0061699	0.004173478	1	1	peptidyl-lysine deglutarylation : BP	BP
GO:0010658	0.004244718	2	24	striated muscle cell apoptotic process : BP	BP
GO:1905099	0.004386961	1	1	positive regulation of guanyl-nucleotide exchange factor activity : BP	BP
GO:2001108	0.004386961	1	1	positive regulation of Rho guanyl-nucleotide exchange factor activity : BP	BP
GO:0072659	0.004445717	4	165	protein localization to plasma membrane : BP	BP
GO:0071444	0.004453429	1	1	cellular response to pheromone : BP	BP
GO:0060560	0.004753765	4	171	developmental growth involved in morphogenesis : BP	BP
GO:0032102	0.004963298	4	169	negative regulation of response to external stimulus : BP	BP
GO:0016049	0.005000554	6	393	cell growth : BP	BP
GO:0071326	0.005189427	3	86	cellular response to monosaccharide stimulus : BP	BP
GO:0071331	0.005189427	3	86	cellular response to hexose stimulus : BP	BP
GO:0048812	0.00566515	6	409	neuron projection morphogenesis : BP	BP
GO:1990778	0.005734383	4	177	protein localization to cell periphery : BP	BP
GO:0046676	0.005734885	2	28	negative regulation of insulin secretion : BP	BP
GO:0061098	0.005736012	2	28	positive regulation of protein tyrosine kinase activity : BP	BP
GO:1903307	0.005819606	2	28	positive regulation of regulated secretory pathway : BP	BP
GO:1902803	0.00583932	2	28	regulation of synaptic vesicle transport : BP	BP
GO:0048841	0.00606868	2	30	regulation of axon extension involved in axon guidance : BP	BP
GO:0031667	0.006093592	5	285	response to nutrient levels : BP	BP
GO:0090278	0.006162074	2	29	negative regulation of peptide hormone secretion : BP	BP
GO:0071322	0.006271035	3	92	cellular response to carbohydrate stimulus : BP	BP
GO:0051241	0.006458135	8	690	negative regulation of multicellular organismal process : BP	BP
GO:1902667	0.006469567	2	31	regulation of axon guidance : BP	BP
GO:0002792	0.006604922	2	30	negative regulation of peptide secretion : BP	BP
GO:0051262	0.007028151	3	95	protein tetramerization : BP	BP
GO:0050919	0.007187646	2	32	negative chemotaxis : BP	BP
GO:0050830	0.007425082	2	31	defense response to Gram-positive bacterium : BP	BP
GO:0051650	0.00746725	4	191	establishment of vesicle localization : BP	BP
GO:0071526	0.00758786	2	33	semaphorin-plexin signaling pathway : BP	BP
GO:0051961	0.007642944	4	193	negative regulation of nervous system development : BP	BP
GO:1903034	0.007710661	3	99	regulation of response to wounding : BP	BP
GO:0048846	0.007758898	2	34	axon extension involved in axon guidance : BP	BP
GO:1902284	0.007758898	2	34	neuron projection extension involved in neuron projection guidance : BP	BP
GO:1900454	0.007777389	1	2	positive regulation of long term synaptic depression : BP	BP
GO:0060733	0.007821373	1	2	regulation of eIF2 alpha phosphorylation by amino acid starvation : BP	BP
GO:0071262	0.007821373	1	2	regulation of translational initiation in response to starvation : BP	BP
GO:0071264	0.007821373	1	2	positive regulation of translational initiation in response to starvation : BP	BP
GO:0045908	0.007997016	1	2	negative regulation of vasodilation : BP	BP
GO:0032229	0.008046974	1	2	negative regulation of synaptic transmission, GABAergic : BP	BP
GO:0039519	0.00819693	1	2	modulation by virus of host autophagy : BP	BP
GO:0007412	0.008269098	1	2	axon target recognition : BP	BP
GO:0061107	0.008271037	1	2	seminal vesicle development : BP	BP
GO:0021644	0.008275038	1	2	vagus nerve morphogenesis : BP	BP
GO:0031630	0.008281182	1	2	regulation of synaptic vesicle fusion to presynaptic membrane : BP	BP
GO:0097156	0.008321807	1	2	fasciculation of motor neuron axon : BP	BP
GO:0055099	0.00835166	1	2	response to high density lipoprotein particle : BP	BP
GO:0047496	0.008360167	2	34	vesicle transport along microtubule : BP	BP
GO:0021784	0.00839696	1	2	postganglionic parasympathetic fiber development : BP	BP
GO:0010660	0.008428257	2	34	regulation of muscle cell apoptotic process : BP	BP
GO:0006904	0.008486159	2	34	vesicle docking involved in exocytosis : BP	BP
GO:2001106	0.008531384	1	2	regulation of Rho guanyl-nucleotide exchange factor activity : BP	BP
GO:0009991	0.008611824	5	310	response to extracellular stimulus : BP	BP
GO:0051648	0.008890114	4	201	vesicle localization : BP	BP
GO:0016233	0.009392664	2	36	telomere capping : BP	BP
GO:0045685	0.009727248	2	37	regulation of glial cell differentiation : BP	BP
GO:1903531	0.009773705	3	108	negative regulation of secretion by cell : BP	BP
GO:0032874	0.009812933	3	109	positive regulation of stress-activated MAPK cascade : BP	BP
GO:0061045	0.009891093	2	37	negative regulation of wound healing : BP	BP

GO Analysis_ Module Lightcyan

GO.category	GO.over_represented_pvalue	GO.numDEInCat	GO.numInCat	GO.term	GO.ontology
GO:2000543	0.001194491	2	4	positive regulation of gastrulation : BP	BP
GO:0050907	0.001195026	3	14	detection of chemical stimulus involved in sensory perception : BP	BP
GO:0007606	0.002488361	4	38	sensory perception of chemical stimulus : BP	BP
GO:0046889	0.004418634	4	44	positive regulation of lipid biosynthetic process : BP	BP
GO:0021694	0.004533287	2	8	cerebellar Purkinje cell layer formation : BP	BP
GO:0021702	0.004533287	2	8	cerebellar Purkinje cell differentiation : BP	BP
GO:0033555	0.004596875	4	46	multicellular organismal response to stress : BP	BP
GO:0006833	0.004777049	2	7	water transport : BP	BP
GO:0048266	0.005252874	2	8	behavioral response to pain : BP	BP
GO:0031338	0.005714037	4	48	regulation of vesicle fusion : BP	BP
GO:0042159	0.005860528	2	8	lipoprotein catabolic process : BP	BP
GO:0001580	0.006697547	2	8	detection of chemical stimulus involved in sensory perception of bitter tast	BP
GO:0015837	0.00728313	3	26	amine transport : BP	BP
GO:0051952	0.00728313	3	26	regulation of amine transport : BP	BP
GO:0007186	0.007978361	13	404	G-protein coupled receptor signaling pathway : BP	BP
GO:1901642	0.008034575	2	9	nucleoside transmembrane transport : BP	BP
GO:0050912	0.008484177	2	9	detection of chemical stimulus involved in sensory perception of taste : BP	BP
GO:0021692	0.008913384	2	11	cerebellar Purkinje cell layer morphogenesis : BP	BP

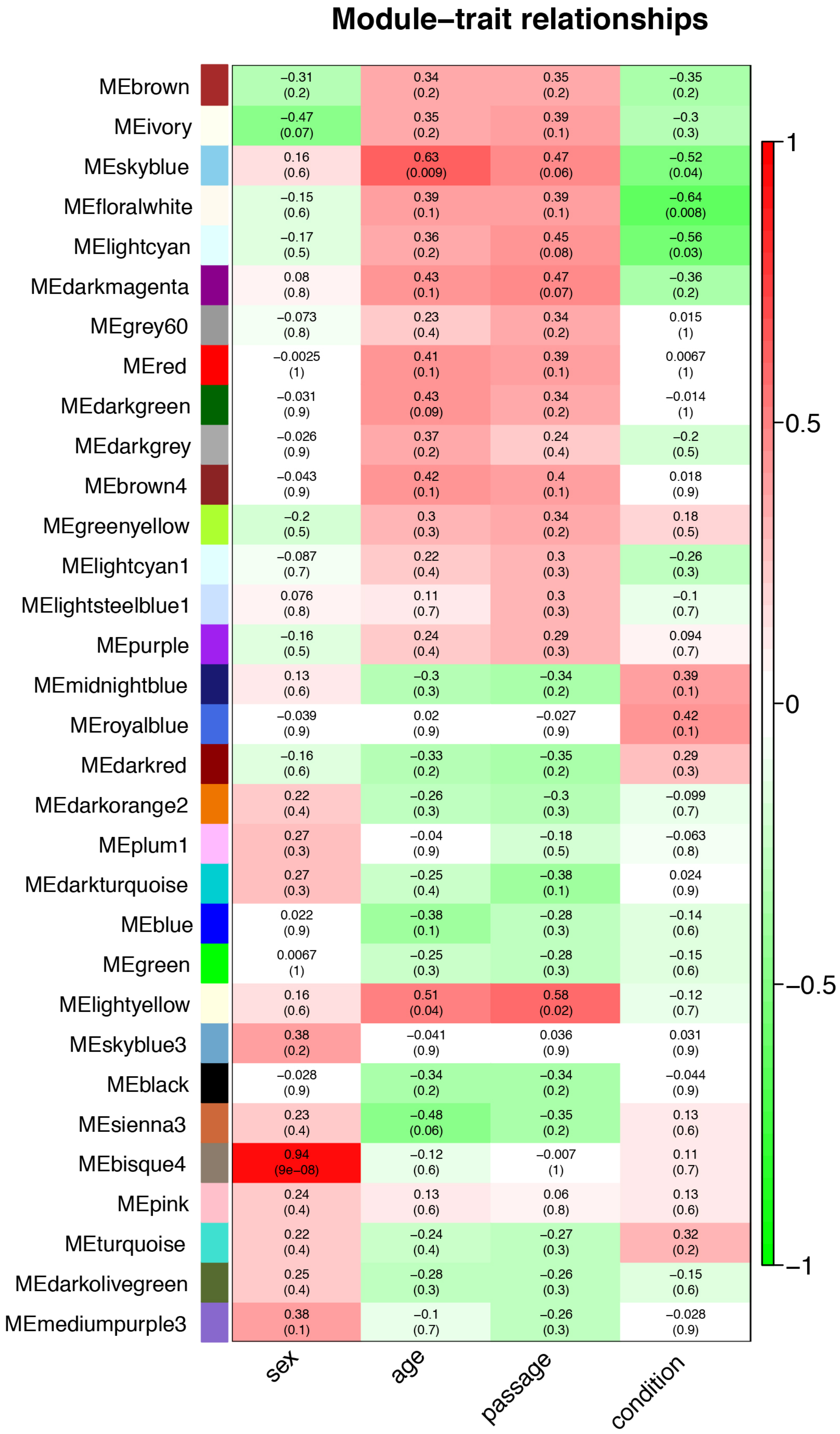

Supplementary Figure S1: Co-expression networks heatmap identified by WGCNA. Each module assigned to a certain color on the left. All considered traits mentioned below the heatmap. Side legend: values of co-expression module for each sex, age, passage and condition trait. P-values are listed in parenthesis below the expression value for each module-trait combination.
